# Clinical Efficacy of CyberKnife Radiosurgery for Adult Brainstem Glioma: 10 Years Experience at Tianjin CyberKnife Center and Review of the Literature

**DOI:** 10.3389/fonc.2019.00257

**Published:** 2019-04-12

**Authors:** Jiaqi Zhang, Qun Liu, Zhiyong Yuan, Lujun Zhao, Xiaoguang Wang, Ping Wang

**Affiliations:** Department of Radiotherapy, National Clinical Research Center of Cancer, Tianjin Medical University Cancer Institute and Hospital, Tianjin, China

**Keywords:** brainstem gliomas, stereotactic body radiotherapy, CyberKnife, radiosurgery, prognostic factor

## Abstract

**Background:** Brainstem glioma is a rare brain tumor with poor prognosis and difficulty for surgical resection. We sought to retrospectively analyze and evaluate the clinical efficacy of CyberKnife for brainstem gliomas.

**Methods:** From 2006 to 2015, a total of 21 brainstem gliomas patients who received CyberKnife radiosurgery treatment enrolled in this study and 18 patients with follow up. CyberKnife image-guided radiosurgical system were applied consecutively with the median prescribed total dose of 26 Gy (14–33 Gy) at two to six fractions on days utilizing CyberKnife system, and the median biological equivalent doses of 59.8 Gy (33.6–76.56 Gy). The clinic pathlogical features, survival were analyzed to explore the efficacy of CyberKnife radiosurgery in treatment of brainstem glioma.

**Results:** With median follow-up of 54.5 months, patients with brainstem gliomas had median overall survival of 19 months, five patients still alive. The primary endpoints of the 1- and 2-year overall survival rates were 87.5 and 52.4%, respectively. During the treatment course, six patients were observed to have pseudoprogression with mass effect on MRI. Four patients developed radiation complications. Grade 2 radiation-related toxicity were observed in three patients and one patient with grade 3.

**Conclusion:** The efficacy of brainstem gliomas—treated with CyberKnife is efficacious with mild toxicity.

## Introduction

Brainstem gliomas (BSGs) account for 5–15% of brain tumors and more likely happen in children (1). BSGs constitute at least 20% of childhood brain neoplasms (2), and the peak age is 7–9 years. In contrast, BSGs is rare in the adult population and account for only 1.5–2.5% of brain glioma, with a peak age of 40–70 years (1). Diffuse intrinsic pontine glioma(DIPG) was the most common type of BSG is that could invade the whole brainstem including midbrain, pons as well as medulla, thus surgery resection has the most challenges and may not be taken as the appropriate approach. In the meanwhile, even some progress have been made recently in genomic profile of DIPG, the prognosis still remains poor, with an average survival of 10–12 months (3). Unlike children, there is a much more broad pathology spectrum in adult brainstem glioma, which squint toward be more radiographically and clinically diversified. The study done by Babu et al. reported that diffuse intrinsic low grade BSGs in adult are the main subgroup of BSGs with a poor prognosis in comparing with malignant BSGs (4).

The main function for brainstem which is composed of brainstem nuclei and white matter tracts is to maintain basic life function such as respiratory as well as heart beat ect, thus, to resect brainstem neoplasm becomes challenging. So does the minimum invasive biopsy procedure which will lead to significant risk for complications (5–7). Consequently, radiotherapy and radiochemotherapy are the primary approaches for BSGs.

With the advent of CyberKnife system (Accuray, Sunnyvale, USA), the frameless image-guided robotic radiosurgery solution, the selected radiation beams could be delivered with submilimeter accuracy. Thus, it can delivers a prescribed dose to the target volume over 1–5 fractions precisely, while doses received by the surrounding normal tissues are maximally reduced. This feature plays a very important role in the treatment for tumors adjacent to critical structures. Cyberknife can also track the motion of tumors, continuously monitor, and correct for error. Compared to conventional radiotherapy, CyberKnife radiosurgical system can tremendously protects the adjacent critical structures and improves prognostic condition of patients with BSG. Furthermore, Cyberknife is a frameless image- guided radiosurgery system, it can reduces uncomfortable of patients.

To the best of our knowledge, few studies on the clinical efficacy of BSGs treated with CyberKnife. Thus, the effectiveness and the adverse reaction were evaluated on the treatment of BSGs by CyberKnife in our study.

## Materials and Methods

### Patient Population

We retrospective analyze 21 consecutive adult patients with brainstem gliomas using CyberKnife between 2006 and 2015. The patient inclusion criteria were as follows: (1) all patients were received the cranial computed tomography (CT) and magnetic resonance imaging (MRI), MRI sequence including T1-weighted, T2-weighted, contrast-enhanced T1-weighted MRI and FLAIR, (2) the tumors were diagnosed as glioma by two or more medical specialists in diagnostic radiology based on the typical CT and MRI findings of such lesions, (3) the epicenter of the tumor was located in the brainstem (midbrain, pons, and medulla oblongata). For differential diagnosing from other type, the patients with diffuse intrinsic tumors (in which the border between the tumor and brainstem was indistinct), cystic tumors and well-delineated dorsally exophytic tumors can be excluded on T2w-MRI due to more suitable for radiotherapy or surgery (8, 9). Clinical data collected contained sex, age, diagnosis, baseline and subsequent neurological symptoms, lesion location. In addition, prescription dose, planning target volume, and concurrent therapy were also obtained.

### Planning and Treatment

Before treatment, all patients underwent an enhanced T1-weighted MRI examinations with slice thickness of 2 mm for diagnostic purposes. In the meanwhile, the planning CT was applied for all patients with slice thickness of 1.5 mm. All these data were put in the treatment planning system. In our study, the gross tumor volume (GTV) was determined based on individualized contrast-enhanced CT images and MRI scans, the planning target volume (PTV) was defined by encompassed 1.6 mm outside the gross tumor volume due to CT were applied with slice thickness of 1.5 mm.

The median tumor volume was 25.44 ml (range 17.81–39.64 ml). The median prescribed dose was 26 Gy (range 14–33 Gy), the median BED were 59.8 Gy (33.6–76.56 Gy), and the dose was delivered in two to six fractions (median: five) depend on tumor volume and physical condition on consecutive days with CyberKnife. The median isodose line is of 80% (72–85%) prescribed to all patients covered the PTV. Biological effective dose (BED) was calculated with α/β ratio of 5 for the tumor and 2 for the brainstem (Table 1). The irradiated volume of brainstem above 26 Gy at most 1 cm^3^. The 31 and 25 Gy are recommended to be the maximum dose of brainstem and optic pathway during five-fraction radiosurgery to reduce the occurrence of any adverse effects of radiation (10, 11). During radiotherapy six patients received temozolomide.

**Table 1:** Treatment scheme.

**Characteristics**	**Case %**
Total tumor volume, median	25.44 (cm3)
Target volume, median	9.95 (cm3)
**DOSE(Gy)**	
≤20	1 (4.8%)
21–30	19 (90.4%)
≥31	1 (4.8%)
**FRACTION**	
≤2	3 (14.3%)
3–4	7 (23.3%)
≥5	11 (52.4%)

### Follow-Up Observation and Evaluation

The follow-ups were considered as starting from the time of the CyberKnife treatment with median of 54.5 months (ranged from 6 to 144 months).

All patients were interviewed and clinically evaluated to update their clinical and personal data and to assess patients' quality of life (QoL). The treatment evaluations included patient history, upper abdomen, and neck B-ultrasonography, brain MRI and contrast CT, hematology, renal, and hepatic function analysis. Follow-ups were planned every 3 months after treatment in the first year. For patients exhibiting complete response (CR), follow-up frequency was every 6 months in the second year and each year thereafter.

Qualitative information about the variation in tumor volume (i.e., decreased, unchanged, or increased) at MRI follow-up was obtained for all patients, except for three of them.

### Statistical Analysis

The overall survival (OS) and median progression-free survival (PFS) were analyzed for 18 patients. OS was defined as the time from the start of treatment to patient death or last follow-up and PFS was defined as the treatment initiation time to first disease progression time or last follow-up time.

Local advancement was determined depend on MRI with contrast enhancement according to Response Evaluation Criteria (RECIST Version 1.1). To confirm pseudoprogression (PsP) occurred after treatment, the serial MRIs was utilized and analyzed. PsP defined as the transient worsening of enhancing abnormalities or mass effect on MRI after radiotherapy. Treatment-related toxicities were evaluated according to Common Terminology Criteria for Adverse Events version 4.03 (CTCAE v4.03). Any new symptoms or increasing neurological symptoms happened after radiotherapy without radiographic disease progression by MRI was deemed radiation-related.

The Kaplan–Meier method was used to calculate the survival, and the results were compared with those of the log-rank test. In multivariate analysis, risk factors for OS from treatment were analyzed by Cox regression model. *P* ≤ 0.05 was considered statistical significance. All statistical analyses were computed by using Stata version 13.1 (StataCorp., 2013. Stata Statistical Software: Release 13.1 College Station, TX, USA: StataCorp LP).

## Results

### Patient Demographics and Tumor Characteristics

Fifteen (71.4%) patients were males, and six (28.6%) ware females. The median age of patients was 39 years (18–70 years). Twenty-one patients had 21 BSGs, and 13 (62%) were located in pontine, 6 (28%) in medullary, 2 (10%) in midbrain. In the meanwhile, six patients were concurrently treated with TMZ for radiochemotherapy (Table 2). All patients' diagnoses were confirmed either by pathology or by typical MRI and MRS.

**Table 2:** Baseline features of patients.

**Characteristics**	**Number of cases (%)**
**SEX**	
Male	16 (71.4%)
Female	5 (28.6%)
Median age(years)	37 (18–70)
**SIGN AND SYMPTOMS PREOPERATIVE**	
Headache	5 (23.8%)
Nausea/vomiting	1 (4.8%)
Hypesthesia	10 (47.6%)
Motor deficits	14 (66.7%)
Cranial nerve palsy	18 (85.7%)
**LESION SITE**	
Midbrain	2 (10%)
Pons	13 (62%)
Medulla	6 (28%)
Concurrent radiochemotherapy	6 (28.6%)
**PSEUDOPROGRESSION**	
Yes	6 (28.6%)
No	15 (71.4%)

### Survival

Of 21 patients, 18 had available follow-up. The median OS was 19 months and five patients are still alive. The 1-, 2-year survival rates were 87.5 and 52.4%, respectively. The median PFS was 15 months and the 1-, 2-year PFS rates were 68.8 and 40.5%, respectively. The headache and nausea suffered by all enrolled patients were controlled; 10 patients (60%) with hypesthesia were eased; 11 patients (78.6%) with motor deficits were alleviated; 15 patients (83.3%) with cranial nerve palsy were relieved. Six patients had PsP. During treatment, four patients were observed to have the radiation complications such as headache, dizziness, and nausea.

**Figure 1:**
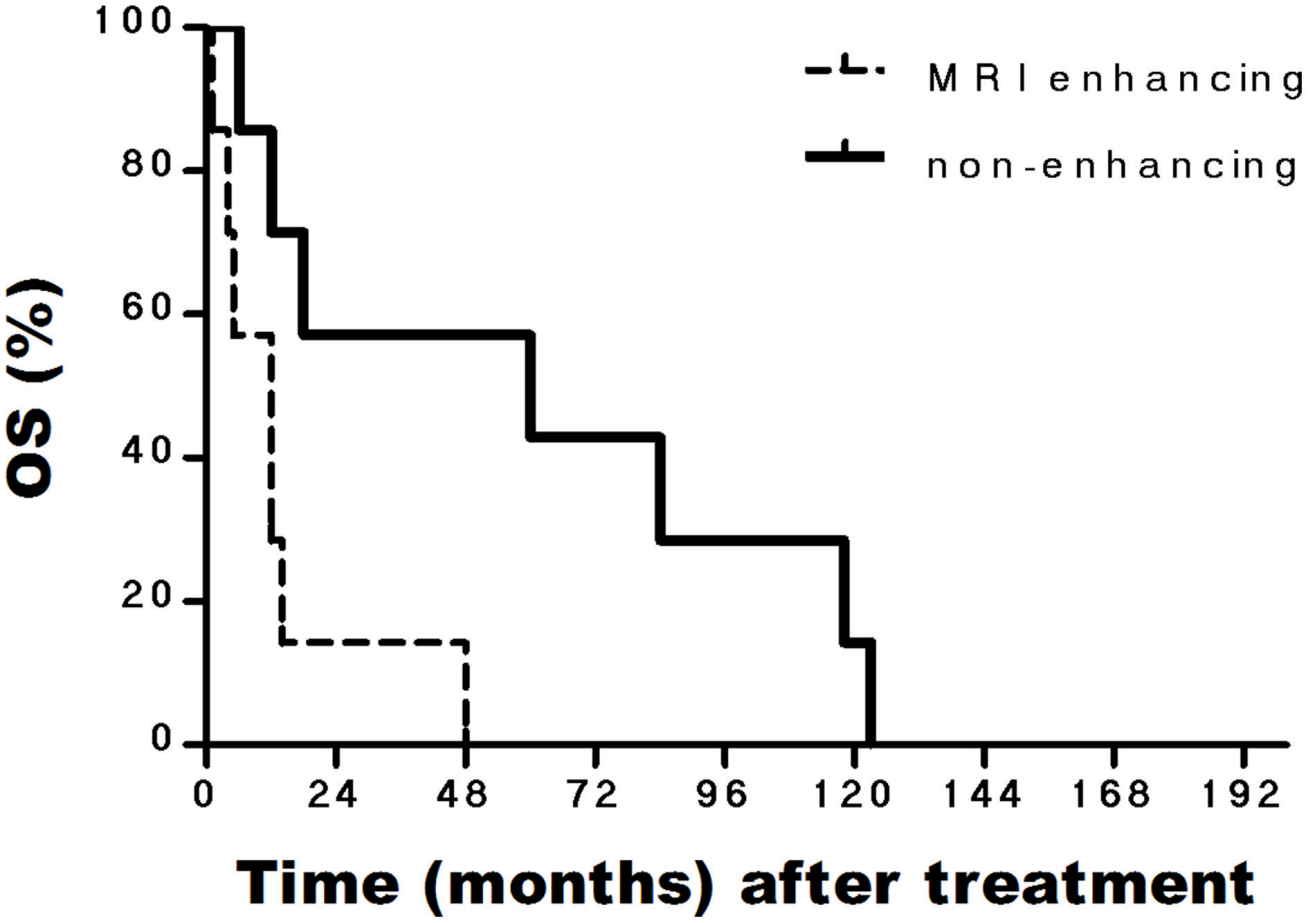
Kaplan-Meier curves of OS based on MRI enhancing and non-enhancing in BSGs patients.

**Figure 2:**
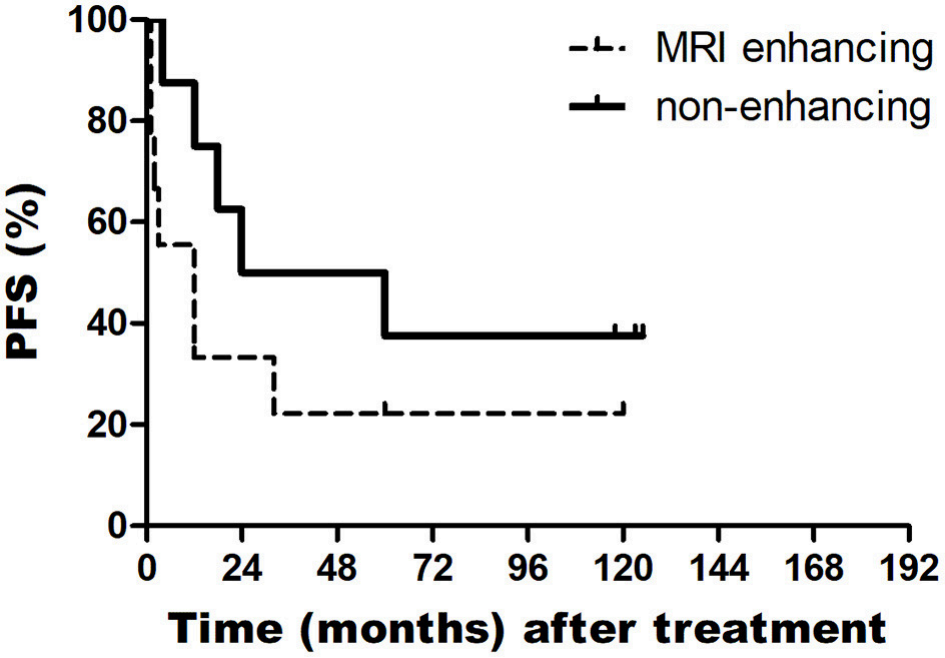
Kaplan-Meier curves of PFS based on MRI enhancing and non-enhancing in BSGs patients.

In addition, the median OS of patients with PsP and without PsP were 60 and 18 months, respectively (*P* = 0.362). The median OS of patients with enhancing tumor in MRI and non-enhancing tumor in MRI were 14 and 60 months, respectively (*P* = 0.043). The median PFS of patients with enhancing tumor in MRI and non-enhancing tumor in MRI were 3 and 24 months, respectively (*P* = 0.088) (Figures 1, 2).

Cox proportional hazards model was used to assessed the variables included sex, age, dose, fractions, tumor volume, pseudoprogression, enhancing tumor in MRI, receipt of concurrent chemotherapy. The multivariate analysis indicted that age and enhancing tumor in MRI were independent prognostic factors of patients with BSGs (Table 3).

**Table 3:** Multivariate prognosis analysis of 18 cases of BSGs.

**Characteristic**	**RR**	**95% CI**	***P*-value**
Sex	0.098	0.009–1.035	0.053
Age	1.096	1.008–1.193	0.032
Dose	1.001	0.999–1.003	0.266
Fractions	0.093	0.880–5.250	0.093
Tumor volume	0.972	0.855–1.106	0.666
Pseudoprogression	0.478	0.092–2.491	0.381
Enhancing tumor in MRI	3.332	0.943–11.776	0.045
Receipt of concurrent chemotherapy	2.339	0.369–14.838	0.367

*MRI, magnetic resonance imaging*.

### Radiotherapy-Associated Toxicity

In our study, four patients (19%) were found radiation complications from CyberKnife treatment. Three patients (14.3%) appeared with grade 2 toxicities, one of them with headache and the others displayed persistent dizziness which were managed with NSAIDS, dexamethasone. And one patient (4.8%) with a grade 3 radiation-related toxicity displayed vomiting and dizziness, the symptoms approximately persisted 1 month after CyberKnife treatment and dexamethasone were prescribed for symptoms remission. The median time to development of symptoms for these patients for the toxicity was 3.4 months (range, 1.6–5.3). We considered the reason of these side effect was brain edema which caused by CyberKnife treatment. Treatment-related toxicities were evaluated using Common Terminology Criteria for Adverse Events version 4.03 (CTCAE v4.03) (Table 4).

**Table 4:** Radiotherapy-associated toxicity.

**Radiotherapy-associated toxicity**	***n* (%)**
Symptomatic radiotherapy-associated toxicity	4(19.0%)
Grade 2 toxicities(headache)	1(4.8%)
Grade 2 toxicities(persistent dizziness)	2(9.5%)
Grade 3 toxicities	1(4.8%)

## Discussion

The brainstem gliomas represent 1.6% of all primary central nervous system (CNS) tumors, 3.6% of CNS tumor, while account for 4.2% in CNS gliomas (3). The morbidity of BSGs in children and adults are different. In pediatric patients, BSGs account for 20% or more of primary glial neoplasms, whereas being rare in adults (1, 2). According to guideline from World Health Organization (WHO), gliomas were classified as Grade I localized gliomas(such as pilocytic astrocytoma) and Grade II to IV infiltrating gliomas such as oligodendrocytic, astrocytic, mixed gliomas, and glioblastomas, all of these maybe detected in the brainstem, as well as have different radiographic features and prognosis.

Due to the location of these tumors and potential influence on the CyberKnife treatment, biopsies are not performed in our series. Most patients enrolled in our study were diagnosed by MRI, MRS features and long time of follow up. MRI is the cornerstone in diagnosing BSGs, the diagnostic accuracy of MRI for brainstem glioma could be up to 95.3% (12), however, histopathological type could not be distinguished by MRI (12, 13). Since the biopsies procedures are hard to implement in our study, the pathological type could not be determined for the all the cases enrolled in our studies, the survival outcomes might be impacted without biopsy result. The surgical morbidity was not a negligible issue, yet, molecular and biological characterization of brainstem glioma may provide a new way in further for making more effective therapeutic regimen.

Brainstem glioma is a rare brain tumor with a poor prognosis. Its clinical symptoms are associated with the location and the way of tumors' growth and infiltration. The most common neurological symptoms include headache, vertigo, paraesthesias, ataxia, and muscle force degrading etc. BSGs are difficult to treat by surgical resection which may cause serious complications and death, because the tumors invade the brainstem. Radiotherapy is a safe and effective modality for brainstem tumor. The prescribed dose in conventional of radiotherapy is about 50–55 Gy with 1.8–2 Gy per fraction and the therapeutic improvement for symptoms relief has been achieved in at least 60% of the patients with BSGs (13). Shrieve and his co-workers have conducted the study in which that 14 children and 9 adults with BSGs were treated with hyperfractionated radiotherapy, the median survival were 190 weeks for adults and 71.7 weeks for children respectively, 1 and 2 year survivals were 68.4 and 52.6% for adults and 63.4 and 32% for children, respectively (14).

Malignant BSGs have a poor prognosis and insensitive to radiotherapy, it was reported that clinic and radiographic response approaches 13% cases (15–18). Simultaneous chemotherapy of TMZ combined with Bevacizumab can be administrated when patients show failure with radiotherapy (13). Boothe et al. reported results for 11 patients with cerebral radiation necrosis (RN), bevacizumab were used for patients with brain metastases who was treated by SRS and subsequently RN over a 3-year period. After mean 33 days from the end of bevacizumab treatment, The mean shrink in volume from imaging examination was 67.1% for enhancing RN volume and 61.3% for no enhancing RN volume (*P* = 0.018) (19).

Radiosurgery is also a treatment option for brainstem glioma. Several studies have reported that tectal gliomas underwent Gamma Knife radiosurgery have good outcomes. Yen et al.'s study on Gamma Knife radiosurgery for metastatic brainstem lesions enrolled 45 patients, the prescription doses ranged from 9 to 25 Gy and the median OS was 11 months (20). Fuchs et al. also reported Gamma Knife is an effective treatment modality for brainstem gliomas with satisfying tumor control and clinical recovery (21). In an early research by Yoshikawa et al. 25 patients with malignant gliomas received the CyberKnife treatment, patients with well-controlled lesions had significantly better prognosis in comparing with uncontrolled cases (39.8 vs. 16.0 months) (22).

In our study, patients with BSGs were treated by CyberKnife, the median survival was 19 months and the one, two-year survival rates were 87.5 and 52.4%, respectively. Eighty percent patients of our series showed a enhancing tumor in MRI that is why our series have a shorter OS. Contrast enhancement are related with significantly shorter survival in this study—median OS in patients with non-enhancing tumors was 60 vs. 14 months for patients with enhancing tumors (*P* < 0.05). Many studies have shown that MRI features of BSGs could predict the pathological features and estimate prognosis (12, 13). For BSGs, the absence of contrast enhancement on MRI was a significant factor associated with more favorable pathology (12).

CyberKnife with its good conformity and sharp dose fall-off could maximize the dose to the target and minimize the dose to the adjacent critical structure. While 3D-conformal radiation therapy and intensity modulated radiation therapy are most common applied techniques in the treatment for BSGs, the therapeutic gain could not be compared with CyberKnife for its accurate dose delivery to the target and minimize the dose to the critical structures such as optic nerves and optic chiasm during gliomas treatment. Different from frame based SRS delivered by Gamma Knife, the frameless CyberKnife could deliver multi-session SRS, thus, it is more suitable for the radiosurgery treatment to the large tumors.

In our study, the mean tumor size is 25.44 ml, and mean ROL volume is 9.95 ml. The lesion size is larger than the published studies. While in Lin et al.'s series, the median tumor volume for 45 brainstem metastases was 0.40 cm^3^, while the median OS was 11.6 months (23). In addition, in El-Shehaby et al.'s series on 11 patients with malignant gliomas, the median tumor volume was 4.5 ml, eight patients occurred neurological deterioration transiently (24). Valery et al. showed on 30 patients with a mean tumor volume of 2.82 ml, the median survival was 10 months (25) (Table 5). Although the median tumor volume in our study is larger compared to the mentioned studies, the OS or prognosis are comparable. These comparable results suggest that increasing tumor volume may not negatively impact survival.

**Table 5:** Summary of studies in which radiotherapy was used for treatment of brainstem gliomas.

**Authors**	**Total no. of cases**	**Treatment**	**ROL volume (ml)**	**Median overall survival (months)**
Liu et al. (26)	66	CyberKnife	0.14	5
El-Shehaby et al. (24)	11	Gamma Knife	4.5	–
Yen et al. (27)	20	Gamma Knife	2.5	–
Jung et al. (28)	32	Gamma Knife	0.71	5.2
Lopez et al. (29)	10	Interstitial radiosurgery	2.76	–
Lin et al. (23)	45	Linac-SRS	0.40	11.6
Valery et al. (25)	30	Linac-radiosurgery	2.82	10
Our study	21	CyberKnife	9.95	19

*linac-SRS, linear accelerator-based stereotactic radiosurgery*.

As described in the previous study, tumor progression and radiation necrosis were hard to identify on imaging. DWI, MRS, PWI, and FET/PET can be applied to distinguish the radiation necrosis from tumor progression (13).

**Figure 3:**
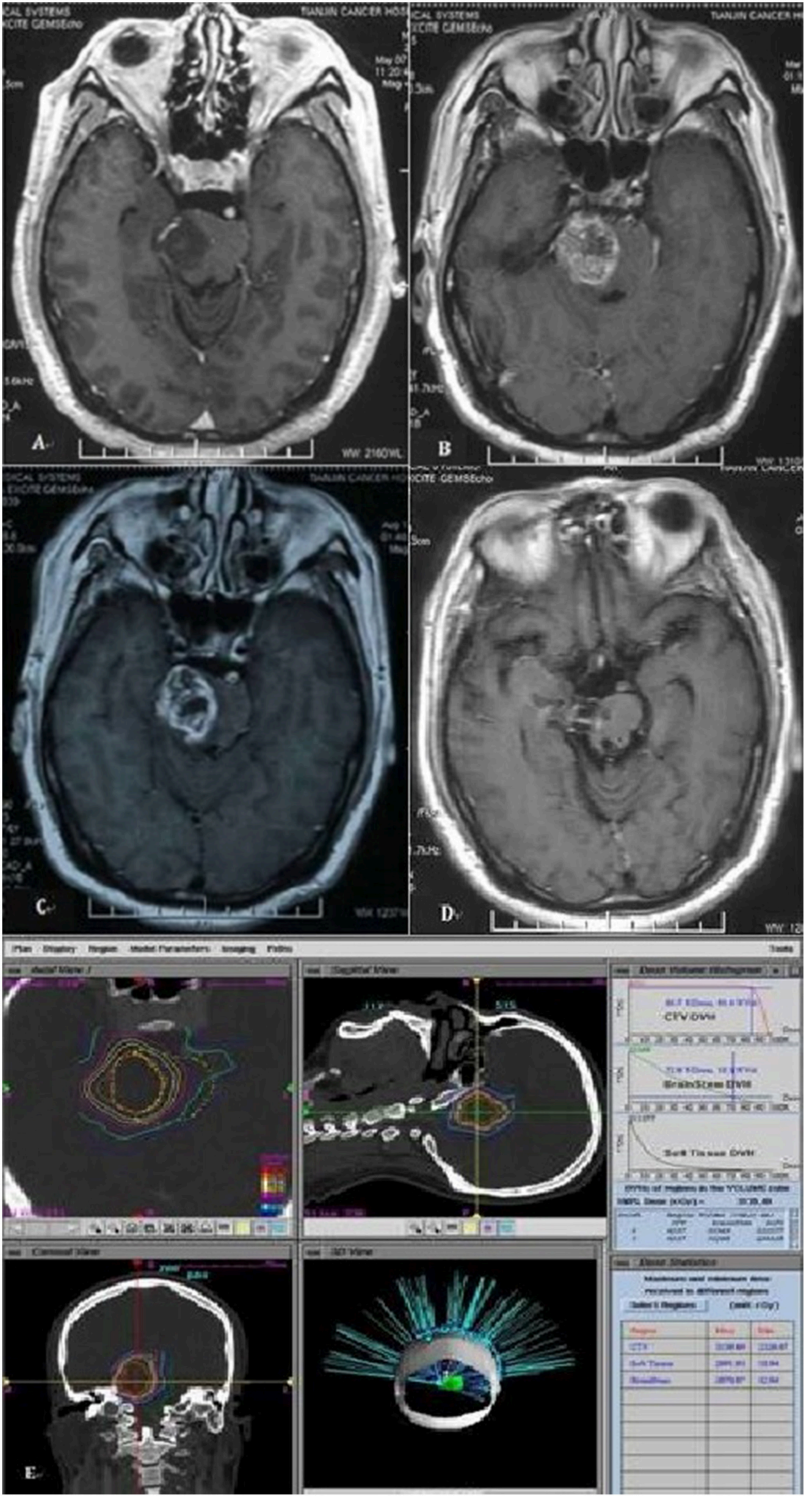
Magnetic resonance image of patient with pseudoprogression. **(A)** MRI of a 45-year-old man with pons glioma diagnosed by MR. **(B)** Eleven months after treatment, pseudoprogression happened on MR. **(C)** Fifteen months after treatment. **(D)** Two year after treatment, pseudoprogression gradually resolution. **(E)** The ROI volume of this patient is 8.2 ml with 2,700 cGy.

PsP may occur in 14–31% of patients with malignant glioma treated by radiotherapy (30–32) and impacted by treatment dose and tumor volume. PsP usually be seen in patients with malignant gliomas after chemoradiotherapy, and occurred 6 months later with the initial treatment, defined as the transient worsening of enhancing abnormalities or mass effect on MRI (Figure 3). The diagnosis with MRS and treatment with DXM and Bevacizumab could be considered. In our study, PsP occurred in six patients (28.6%) after treatment and relapsed in the area with high dose, the patients were with median OS of 60 months while another 15 patients without PsP enrolled in our study were with median OS of 17 months. OS of patients with PsP were longer than the patients which didn't occur PsP, even the statistical analysis was with *P* > 0.05. The reason of this result maybe the limitation of our study is the small sample size.

## Conclusions

The treatment of BSGs with CyberKnife is suggested to be effective. Our study shows the increased patients' symptoms relief rates with brainstem BSGs treatment by CyberKnife. The treatment accuracy, the extent of dose injury to the adjacent critical structure and the adequate target doses converge are vital to the therapeutic effect in which CyberKnife achieved high performance.

## Ethics Statement

Tianjin Cancer Institute and Hospital ethics committee approved the study. Written informed consent was obtained from the patients for their participation.

## Author Contributions

JZ, QL, and PW are the main authors of the manuscript, and have made substantial contributions to the conception and design of the study. The sample collection was performed by XW, LZ, and ZY gave the final approval and revision of the manuscript. All authors read and approved the final manuscript.

### Conflict of Interest Statement

The authors declare that the research was conducted in the absence of any commercial or financial relationships that could be construed as a potential conflict of interest.

## Abbreviations

GBSs: brainstem gliomas
DIPG: diffuse intrinsic pontine glioma
OS: overall survival
BED: biological equivalent doses
MRI: magnetic resonance imaging
GTV: gross tumor volume
PTV: planning target volume
PH: proportional hazards
CNS: central nervous system
WHO: World Health Organization
RN: radiation necrosis
PsP: Pseudoprogression
linac-SRS: linear accelerator-based stereotactic radiosurgery.
